# Analysis of gut and circulating microbiota characteristics in patients with liver cirrhosis and portal vein thrombosis

**DOI:** 10.3389/fmicb.2025.1597145

**Published:** 2025-06-19

**Authors:** Ping Qi, Xu-xu Yang, Cun-kai Wang, Wei Sang, Wei Zhang, Yun Bai

**Affiliations:** ^1^Graduate School, Hebei North University, Zhangjiakou, China; ^2^Department of Agedness Gastroenterology, Hebei General Hospital, Shijiazhuang, China

**Keywords:** liver cirrhosis, portal vein thrombosis, gut microbiota, circulating microbiota, microbial translocation

## Abstract

**Background:**

Portal vein thrombosis (PVT) is a common and serious complication of liver cirrhosis, often associated with worsened prognosis and increased risk of hepatic decompensation. The role of gut and circulating microbiota in its pathogenesis remains unclear.

**Methods:**

We enrolled cirrhotic patients with PVT (*n* = 17) and cirrhotic patients without PVT (*n* = 25). Fecal and peripheral blood samples were collected from all; portal vein samples were obtained from 16 patients undergoing TIPS. 16S rRNA sequencing was performed on fecal, peripheral blood, and portal venous blood samples to compare the diversity, structural differences, key microbial taxa, and characteristic variations of gut and circulating microbiota between cirrhotic patients with and without PVT.

**Results:**

(1) Gut microbiota showed no α-diversity difference between groups, but β-diversity differed significantly. PVT patients had increased Gram-negative bacteria (such as *Escherichia-Shigella*) and decreased SCFA-producing taxa. (2) Compared with peripheral vein microbiota, portal vein microbiota showed significant difference in α diversity and β diversity in cirrhotic patients with PVT, with *Massilia* enriched. (3) Portal microbiota had the highest diagnostic value for PVT (AUC = 0.95). (4) The tPVT group had more portal-feces shared genera than the tNPVT group (49 vs. 29). Portal-peripheral-feces shared taxa were predominantly LPS-producing Gram-negative bacteria such as *Escherichia-Shigella* and *Klebsiella*. (5) Most bacterial genera in the portal vein showed significant positive correlations with LPS and FVIII in the portal vein. Genera such as *Faecalibacterium*, *Eubacterium_hallii_group*, *Ruminococcus*, *Agathobacter*, *Bacteroides*, and *Romboutsia* were significantly negatively correlated with Child-Pugh scores. *Faecalibacterium*, *Eubacterium_hallii_group*, *Alistipes*, *Ruminococcus*, *Agathobacter*, *Bacteroides*, *Blautia*, and *Subdoligranulum* were significantly negatively correlated with MELD scores. *Ruminococcus* and *Agathobacter* were significantly negatively correlated with D-Dimer, while *Subdoligranulum* showed significant positive correlations with LPS and FVIII in the portal vein.

**Conclusion:**

Intestinal dysbiosis and translocation in cirrhotic patients with PVT lead to differential changes in the portal and peripheral circulatory microbiomes. This may contribute to the formation of PVT by inducing endotoxemia and systemic inflammation, providing a new microbiological perspective on the pathogenesis of cirrhosis-related PVT through the gut-liver axis.

## 1 Introduction

PVT is a vascular disorder involving the main portal vein and its branches, characterized by thrombus formation and extension within the portal venous system. The thrombus may propagate into the splenic or mesenteric veins, leading to varying degrees of luminal narrowing or complete occlusion (Hasan et al., 2020). The prevalence of PVT in patients with liver cirrhosis ranges from 0.6% to 16% (Senzolo et al., 2019). As a severe complication of cirrhosis, PVT exacerbates portal hypertension, increasing the risk of esophagogastric variceal bleeding and refractory ascites while reducing liver transplantation success rates and elevating mortality (Qi et al., 2015; Stine et al., 2015). The underlying mechanisms of PVT remain unclear. This study focuses on the pathogenesis of PVT, aiming to identify key factors involved in its development and provide new clinical insights for improving the prognosis of cirrhotic patients.

The pathogenesis of PVT is complex and is currently believed to result from the interplay of multiple factors, including reduced portal venous blood flow velocity, coagulation disorders, endothelial injury, and inflammatory responses (Anton et al., 2022; Huang et al., 2023). Multiple studies (Violi and Ferro, 2013; Carnevale et al., 2017; Praktiknjo et al., 2020) have shown that LPS levels in the portal circulation of cirrhotic patients are significantly higher than those in the systemic circulation. Within the portal venous system, LPS binds to Toll-like receptors (TLRs) expressed on hepatocytes and immune cells, triggering the release of large amounts of inflammatory cytokines, chemokines, vasoactive factors, adhesion molecules, and reactive oxygen species (ROS) (Custodio-Chable et al., 2020). This cascade promotes systemic inflammation, hepatic stellate cell proliferation, and the progression of liver cirrhosis (Tsuchida and Friedman, 2017). Additionally, LPS interacts with TLRs on vascular endothelial cells, platelets, and neutrophils, initiating a systemic inflammatory response and activating the coagulation cascade (Hasan et al., 2020). LPS induces oxidative stress and apoptosis in vascular endothelial cells, impairing their anticoagulant function. Endothelial dysfunction reduces nitric oxide (NO) synthesis, exacerbating vasoconstriction and worsening portal venous stasis (Bartimoccia et al., 2024).

In recent years, with the expanding field of microecology, increasing attention has been given to gut microbiota dysbiosis and its role in the pathological processes of liver cirrhosis. Studies have shown that patients with liver cirrhosis commonly exhibit significant gut microbiota dysbiosis, characterized by a marked reduction in beneficial bacteria (e.g., *Bifidobacterium* and *Bacteroidetes*) and a notable increase in opportunistic pathogens (e.g., *Enterobacteriaceae* and *Fusobacterium*) (Bajaj et al., 2014; Oh et al., 2020). The increased abundance of Gram-negative bacilli leads to elevated production of LPS in the intestinal lumen. Through mechanisms involving intestinal barrier dysfunction (gut leakage), inflammatory activation, endothelial injury, coagulation abnormalities, and hemodynamic alterations, LPS contributes to the development of PVT.

Portal venous blood serves as a critical conduit for gut microbiota entry into the systemic circulation, and its microbial composition can provide insights into the microbiological mechanisms underlying PVT. Previous studies have primarily focused on fecal microbiota, while research on circulating microbiota remains limited. This study innovatively employs 16S rRNA sequencing to systematically compare the gut, peripheral blood, and portal venous microbiota in cirrhotic patients with PVT. By analyzing microbial composition, diversity, and their associations with clinical parameters, we aim to elucidate gut-liver axis microbiome characteristics and their role in PVT development. Our findings will provide microbiological evidence for the systemic inflammation associated with PVT and offer novel perspectives on its pathogenesis.

## 2 Materials and methods

### 2.1 Study population

Between November 2023 and November 2024, 42 patients with liver cirrhosis who met the inclusion criteria were recruited at Hebei General Hospital, including 17 with PVT (PVT group) and 25 without PVT (NPVT group). Among them, 16 patients underwent transjugular intrahepatic portosystemic shunt (TIPS), comprising 6 from the PVT group (tPVT group) and 10 from the NPVT group (tNPVT group). The inclusion criteria were: (1) age ≥ 18 years; (2) diagnosis of liver cirrhosis confirmed by clinical, imaging, or histological findings; and (3) diagnosis of PVT confirmed by Doppler ultrasound, contrast-enhanced CT, or MRI. Exclusion criteria included: Use of antibiotics, proton pump inhibitors (PPIs), probiotics, or prebiotics within the past month; Presence of malignancies; Coexisting hematologic disorders; Budd-Chiari syndrome or PVT due to non-cirrhotic causes; Severe infections. Current use of anticoagulants; History of TIPS or liver transplantation; This study was approved by the Medical Ethics Committee of Hebei Provincial People’s Hospital (Approval No. 2022140). All participants received detailed information about the study and provided written informed consent.

### 2.2 Sample collection

All study participants provided a 3-gram fecal sample collected using a sterile standard collector upon admission. Additionally, 5 ml of peripheral venous blood was drawn from the antecubital vein into an EDTA anticoagulant tube. For patients undergoing TIPS, 5 ml of portal venous blood was also collected intraoperatively into an EDTA anticoagulant tube. All samples were immediately frozen at −80°C within 10 min of collection and stored until DNA extraction.

### 2.3 Sequencing methods and bioinformatics analysis

Genomic DNA from fecal and blood samples was extracted using the FastPure Stool DNA Isolation Kit (Vazyme, China). To minimize contamination during blood microbiota analysis, all DNA extractions were performed in a laminar flow cabinet using sterile, DNA-free reagents and consumables. Negative controls, including extraction blanks and PCR no-template controls, were included in each batch to monitor potential contamination.

The 16S V3–V4 region of the genomic DNA was amplified via PCR using the specific primers 338F (5′-ACTCCTACGGGAGGCAGCAG-3′) and 806R (5′-GGACTACHVGGGTWTCTAAT-3′). Library preparation was performed using the NEXTFLEX Rapid DNA-Seq Library Prep Kit, and sequencing was carried out on the Illumina NextSeq 2000 platform with paired-end sequencing (PE300). During the bioinformatics pipeline, sequence clustering was performed using the USEARCH algorithm with a 97% similarity threshold to define amplicon sequence variants (ASVs). The sequences were then aligned and annotated against the Silva (v138) database to obtain species-level information. ASVs detected in negative controls or known reagent contaminants (based on published contamination databases) were removed prior to downstream analyses.

### 2.4 Statistical methods

Statistical analysis was performed using SPSS 26.0 for descriptive and inferential statistics. Normally distributed continuous data were expressed as mean ± standard deviation (*x* ± *s*), and group differences were compared using the independent samples *t*-test. Non-normally distributed data were presented as median (P25–P75), and differences between groups were assessed using the Mann–Whitney U test for non-parametric analysis. For microbiome sequencing analysis, multiple hypothesis testing was conducted using LEfSe, the Wilcoxon rank-sum test (Alpha analysis), ANOSIM (Beta analysis), the Spearman test (Correlation analysis) and Pheatmap methods. The *P*-values of heatmap analysis differing between groups were calculated by Welch’s *t*-test and corrected using the Benjamini–Hochberg false discovery rate (FDR). A *p*-value of < 0.05 was considered statistically significant.

## 3 Results

### 3.1 General clinical data of study population

A total of 42 cirrhotic patients were enrolled, including 17 in the PVT group and 25 in the NPVT group. There was a significant difference in D-Dimer levels between the two groups (*P* = 0.002) (Table 1). Among them, 6 patients underwent TIPS in the tPVT group, and 10 patients underwent TIPS in the tNPVT group. No statistically significant differences were observed in the general clinical data between the tPVT and tNPVT groups (*P* > 0.05) (Table 2).

**TABLE 1:** Comparison of general clinical data between the PVT group and the NPVT group.

Features	PVT (*n* = 17)	NPVT (*n* = 25)	*P*
Gender, (*n*[%])			0.657
Male	12 (70.59)	16 (64.00)	
Female	5 (29.41)	9 (36.00)	
Age (years)	62.12±15.72	56.52±11.40	0.188
BMI (kg/m^2^)	24.93±4.17	24.89±5.09	0.978
Alb (g/L)	30.54±6.07	32.70±6.01	0.261
TB (μmol/L)	25.80 (19.80, 35.80)	25.50 (20.60, 33.20)	0.858
ALT (U/L)	25.60 (14.30, 43.80)	19.60 (15.30, 31.10)	0.617
AST (U/L)	43.30 (24.00, 72.30)	27.70 (22.30, 39.20)	0.330
SCr (μmol/L)	67.40 (52.40, 71.90)	63.70 (52.20, 73.70)	0.599
WBC (10^9^/L)	3.37 (2.73, 5.85)	3.49 (2.54, 5.07)	0.949
HGB (g/L)	107.00 (71.00, 128.00)	83.00 (72.00, 98.00)	0.104
PLT (10^9^/L)	91.00 (60.00, 121.00)	74.00 (55.00 115.00)	0.617
PT (s)	14.00 (13.40, 15.10)	13.40 (12.90, 14.70)	0.336
INR	1.21 (1.15, 1.33)	1.18 (1.10, 1.28)	0.427
FIB (g/L)	2.29±0.92	2.01±0.66	0.253
D-Dimer (mg/L)	4.27 (2.67, 6.96)	1.32 (0.41, 2.62)	0.002
IL-6 (pg/ml)	16.70 (10.00, 60.00)	5.00 (4.20, 27.50)	0.053
IL-8 (pg/ml)	4.80 (3.70, 7.50)	5.90 (3.90, 7.70)	0.538
TNF-α (pg/ml)	4.00 (3.90, 5.40)	4.00 (3.50, 4.00)	0.333
Child-Pugh grade	7.00 (7.00, 9.00)	7.00 (5.00, 9.00)	0.311
MELD grade	11.34 (8.73, 12.51)	10.09 (9.16, 12.25)	0.742

**TABLE 2:** Comparison of general clinical data between the tPVT group and the tNPVT group.

Features	tPVT (*n* = 6)	tNPVT (*n* = 10)	*P*
Gender, (*n*[%])			0.345
Male	5 (83.33)	6 (60.00)	
Female	1 (16.67)	4 (40.00)	
Age (years)	59.50±10.86	58.60±9.44	0.864
BMI (kg/m^2^)	24.23±2.98	22.68±3.42	0.384
Alb (g/L)	35.03±5.94	32.90±7.29	0.555
TB (μmol/L)	20.90 (19.28, 22.30)	24.70 (20.30, 26.58)	0.181
ALT (U/L)	30.10±13.44	28.96±19.77	0.903
AST (U/L)	45.75 (28.27, 66.82)	27.60 (18.98, 50.08)	0.428
SCr (μmol/L)	61.10 (53.00, 69.42)	60.70 (50.70, 67.95)	1.000
WBC (10^9^/L)	3.12 (2.71, 5.49)	3.64 (2.79, 6.64)	0.713
HGB (g/L)	82.00±12.02	83.20±11.21	0.843
PLT (10^9^/L)	105.33±48.65	91.70±40.80	0.556
PT (s)	13.02±1.27	13.51±1.18	0.445
INR	1.13±0.11	1.19±0.12	0.298
FIB (g/L)	2.32±0.54	2.13±0.77	0.599
D-Dimer (mg/L)	3.35 (2.42, 4.21)	1.54 (0.59, 4.41)	0.313
IL-6 (pg/ml)	11.90 (5.88, 49.20)	5.00 (4.25, 25.88)	0.302
IL-8 (pg/ml)	6.20 (4.82, 9.82)	5.45 (4.07, 15.28)	0.713
TNF-α (pg/ml)	4.00 (3.78, 5.12)	4.00 (3.92, 4.10)	0.783
Child-Pugh grade	6.83±0.98	6.80±1.87	0.964
MELD grade	50.95±3.48	45.08±6.13	0.052

### 3.2 Genetic sequencing results of fecal and blood samples

A total of 42 fecal and 58 blood samples were collected, yielding 2,535,401 and 1,813,953 high-quality raw reads, respectively. After sequence processing, 8,130 and 6,712 ASVs were obtained from fecal and blood samples, respectively. Rarefaction curves based on the Shannon index indicated that the sequencing depth was adequate and the experimental results were highly reliable (Supplementary Figures 1A–C).

### 3.3 Gut microbiota characteristics in the PVT and NPVT groups

**FIGURE 1:**
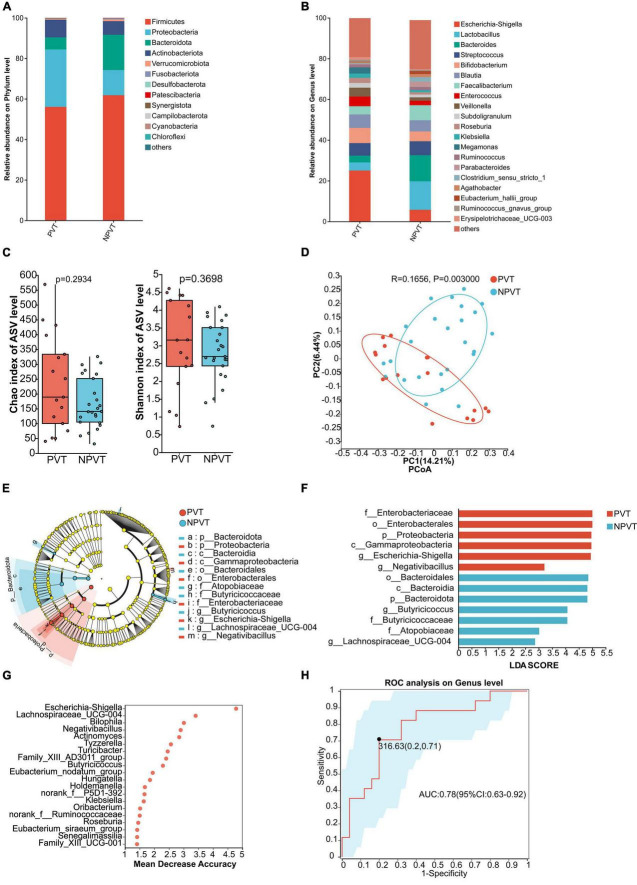
Characterization of fecal bacterial communities in the PVT and NPVT groups. **(A)** Bacterial community composition (phylum level). **(B)** Bacterial community composition (genus level). **(C)** Alpha diversity of bacterial communities between groups based on Chao index and Shannon index. **(D)** PCoA based on Bray–Curtis distance. **(E)** LEfSe results in a cladogram of gut microbiota. **(F)** The histogram of LEfSe analysis reveals differential bacteria (LDA score > 2). **(G)** Random forest model of Top 20 bacterial genera contributing most to the accuracy of predicting PVT groups. **(H)** Bacterial genera used to diagnose PVT in cirrhosis with an AUC of 0.78.

The relative abundance at the phylum level of gut microbiota in both groups showed that the predominant phyla were *p_Firmicutes*, *p_Proteobacteria*, *p_Actinobacteriota*, and *p_Bacteroidota*. Compared to the NPVT group, the PVT group exhibited an increase in *p_Proteobacteria* and *p_Actinobacteriota*, while *p_Firmicutes* and *p_Bacteroidota* were significantly reduced (Figure 1A). At the genus level, the relative abundance of gut bacteria in each group was assessed. In the PVT group, the top five genera were *g_Escherichia-Shigella*, *g_Bifidobacterium*, *g_Blautia* (6.63%), *g_Streptococcus*, and *g_Enterococcus*. In contrast, the top five genera in the NPVT group were *g_Lactobacillus* (14.00%), *g_Bacteroides* (12.81%), *g_Faecalibacterium* (7.40%), *g_Streptococcus* (6.84%), and *g_Escherichia-Shigella* (5.75%) (Figure 1B). At the genus level, there were no statistically significant differences in alpha diversity between the PVT and NPVT groups, as assessed by the Chao index and Shannon index (*P* > 0.05) (Figure 1C). However, the beta diversity analysis based on Bray–Curtis dissimilarity using PCoA revealed significant differences between the two groups (*P* = 0.003) (Figure 1D). LEfSe analysis revealed significant differences in microbial composition between the PVT and NPVT groups. In PVT patients, beneficial commensal bacteria involved in short-chain fatty acid production—such as *Butyricicoccus*, *Lachnospiraceae_UCG-004*, and members of the phylum *Bacteroidota*—were markedly reduced. In contrast, the PVT group showed a significant enrichment of *Proteobacteria*, including *Escherichia-Shigella*, *Negativibacillus*, as well as taxa within *Enterobacteriaceae*, *Enterobacterales*, and *Gammaproteobacteria* (*P* < 0.05, LDA > 2) (Figures 1E, F). The random forest model further evaluated the importance of bacterial genera, revealing that *Escherichia-Shigella*, *Lachnospiraceae_UCG-004*, *Bilophila*, *Negativibacillus*, and *Actinomyces* ranked among the top in feature importance within the model (Figure 1G). To evaluate whether the selected microbiota could effectively discriminate between the two groups, ROC curves based on the top 4 differential microbiota were constructed as biomarkers (Figure 1H). The Area Under the Curve (AUC) reached 0.78 (95% CI: 0.73–0.92), indicating that the gut microbiota biomarkers have good discriminatory power in distinguishing between the PVT and NPVT groups.

### 3.4 Peripheral venous microbiota characteristics in the PVT and NPVT groups

**FIGURE 2:**
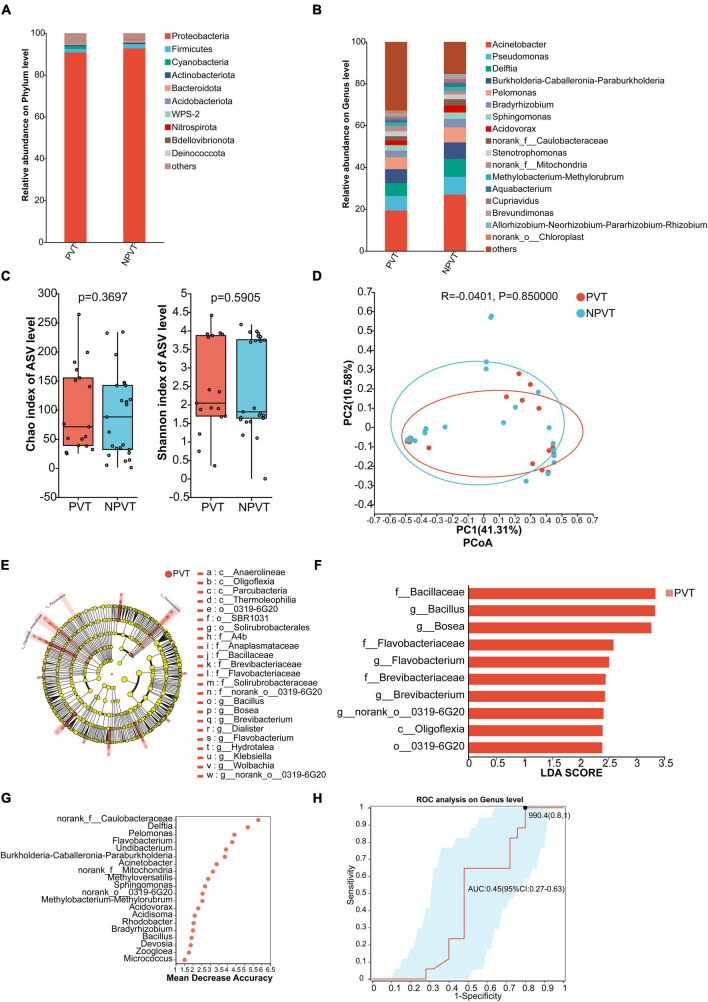
Characterization of Peripheral venous blood bacterial communities in the PVT and NPVT Groups. **(A)** Bacterial community composition (phylum level). **(B)** Bacterial community composition (genus level). **(C)** Alpha diversity of bacterial communities between groups based on Chao index and Shannon index. **(D)** PCoA based on Bray–Curtis distance. **(E)** LEfSe results in a cladogram of gut microbiota. **(F)** The histogram of LEfSe analysis reveals differential bacteria (LDA score > 2). **(G)** Random forest model of Top 20 bacterial genera contributing most to the accuracy of predicting PVT groups. **(H)** Bacterial genera used to diagnose PVT in cirrhosis with an AUC of 0.45.

At the phylum level, both groups showed that *p_Proteobacteria* dominated the peripheral venous microbiota, accounting for more than 90%. The PVT group had a higher abundance of *p_Cyanobacteria* compared to the NPVT group, while *p_Firmicutes*, *p_Actinobacteriota*, and *p_Bacteroidota* were all lower in the PVT group than in the NPVT group (Figure 2A). At the genus level, the relative abundance of peripheral venous bacteria in each group was assessed. In the PVT group, the top five genera were *g_Acinetobacter* (19.35%), *g_Pseudomonas* (6.95%), *g_Burkholderia-Caballeronia-Paraburkholderia* (6.70%), *g_Delftia* (6.18%), and *g_Pelomonas* (4.89%). In the NPVT group, the top five genera were *g_Acinetobacter* (26.96%), *g_Pseudomonas* (8.56%), *g_Delftia* (8.54%), *g_Burkholderia-Caballeronia-Paraburkholderia* (7.86%), and *g_Pelomonas* (7.17%) (Figure 2B). At the genus level, no statistically significant differences were observed in the α diversity or β diversity of the peripheral venous microbiota between the PVT and NPVT groups (*P* > 0.05) (Figures 2C, D). LEfSe analysis revealed that the following taxa were significantly enriched in the PVT group: *f_Bacillaceae*, *g_Bacillus*, *g_Bosea*, *f_Flavobacteriaceae*, *g_Flavobacterium*, *f_Brevibacteriaceae*, *g_Brevibacterium*, *g_norank_o_0319-6G20*, *c_Oligoflexia*, and *o_0319-6G20* (*P* < 0.05, LDA > 2) (Figures 2E, F). Using random forest analysis, the top 20 differential microbiota between the PVT and NPVT groups were selected (Figure 2G). ROC curves based on the top 5 differential microbiota were constructed as biomarkers (Figure 2H). The AUC was 0.45 (95% CI: 0.27–0.63), indicating that the peripheral venous microbiota biomarkers have poor discriminatory power in distinguishing between the PVT and NPVT groups.

### 3.5 Portal venous blood microbiota characteristics in tPVT and tNPVT groups

**FIGURE 3:**
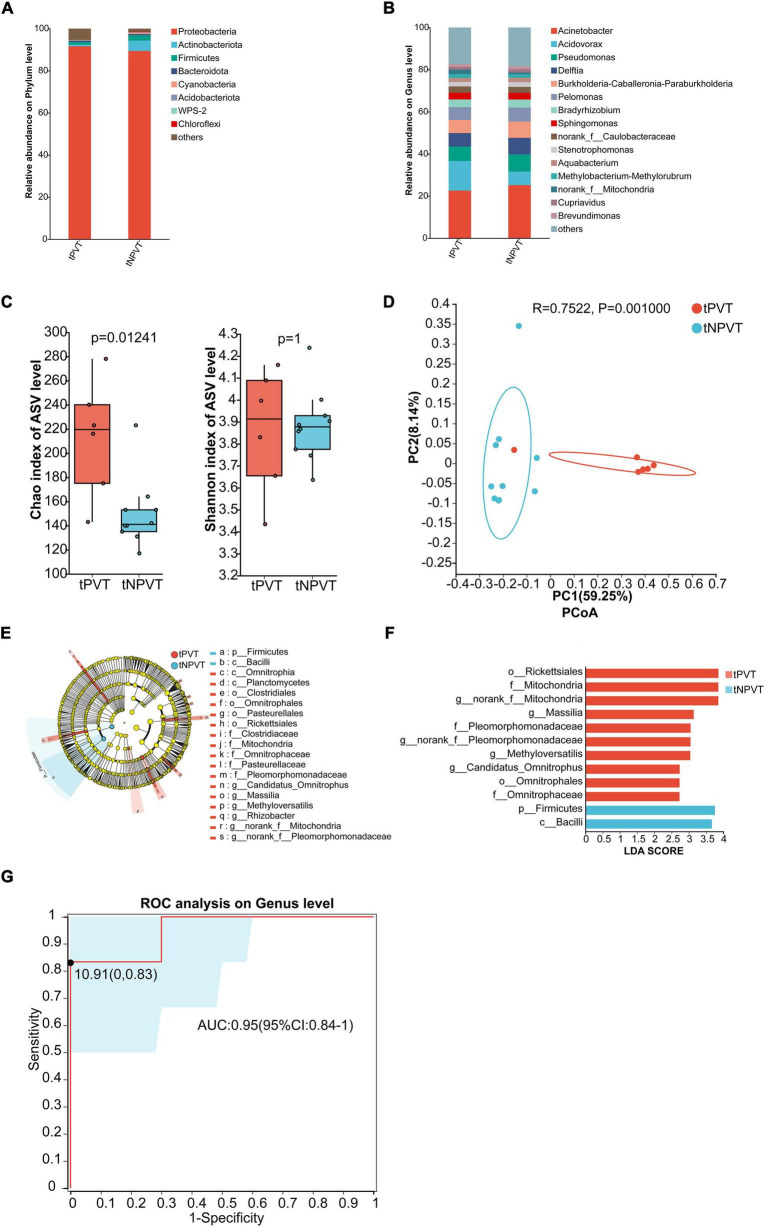
Characterization of Portal venous blood bacterial communities in the tPVT and tNPVT Groups. **(A)** Bacterial community composition (phylum level). **(B)** Bacterial community composition (genus level). **(C)** Alpha diversity of bacterial communities between groups based on Chao index and Shannon index. **(D)** PCoA based on Bray–Curtis distance. **(E)** LEfSe results in a cladogram of gut microbiota. **(F)** The histogram of LEfSe analysis reveals differential bacteria (LDA score > 2). **(G)** Genera were selected based on LEfSe results to diagnose PVT in cirrhosis with an AUC of 0.95.

At the phylum level, the portal venous microbiota in both groups was predominantly composed of *Proteobacteria*. In the tPVT group, *p_Bacteroidota* was slightly higher than in the tNPVT group, while *p_Firmicutes*, *p_Actinobacteriota*, *p_Cyanobacteria*, and *p_Acidobacteriota* were all lower in the tPVT group compared to the tNPVT group (Figure 3A). At the genus level, the relative abundance of bacterial genera in the portal venous blood was assessed for both groups. In the tPVT group, the top five dominant genera were *g_Acinetobacter* (22.59%), *g_Acidovorax* (14.06%), *g_Pseudomonas* (6.87%), *g_Delftia* (6.37%), and *g_Burkholderia-Caballeronia-Paraburkholderia* (6.23%). In the tNPVT group, the top five genera were *g_Acinetobacter* (25.20%), *g_Pseudomonas* (8.25%), *g_Delftia* (7.79%), *g_Burkholderia-Caballeronia-Paraburkholderia* (7.75%), and *g_Pelomonas* (6.62%) (Figure 3B). At the genus level, the α-diversity of the portal venous microbiota, as measured by the Chao index, showed a significant difference between the tPVT and tNPVT groups (*P* < 0.05). However, no significant difference was observed in the Shannon index (*P* = 0.01241) (Figure 3C). Additionally, the β-diversity between the two groups was significantly different (*P* = 0.001) (Figure 3D). LEfSe analysis revealed that in the tPVT group, the significantly enriched bacterial taxa included *o_Rickettsiales*, *f_Mitochondria*, *g_norank_f_Mitochondria*, *g_Massilia*, *f_Pleomorphomonadaceae*, *g_norank_f_Pleomorphomonadaceae*, *g_Methyloversatilis*, *g_Candidatus_Omnitrophus*, *o_Omnitrophales*, and *f_Omnitrophaceae*. In contrast, *p_Firmicutes* and *c_Bacilli* were significantly reduced (*P* < 0.05, LDA > 2) (Figures 3E, F). LEfSe analysis identified the following genera with significant differences at the genus level: *g_Massilia*, *g_Methyloversatilis*, and *g_Candidatus_Omnitrophus*. An ROC curve based on these microbial biomarkers was constructed, and the AUC reached 0.95 (95% CI: 0.84–1) (Figure 3G). This indicates that these three microbes in the portal venous blood have high accuracy in predicting cirrhosis-associated PVT.

### 3.6 The shared microbiota between the tPVT and tNPVT groups in the gut, peripheral venous blood, and portal venous blood

**FIGURE 4:**
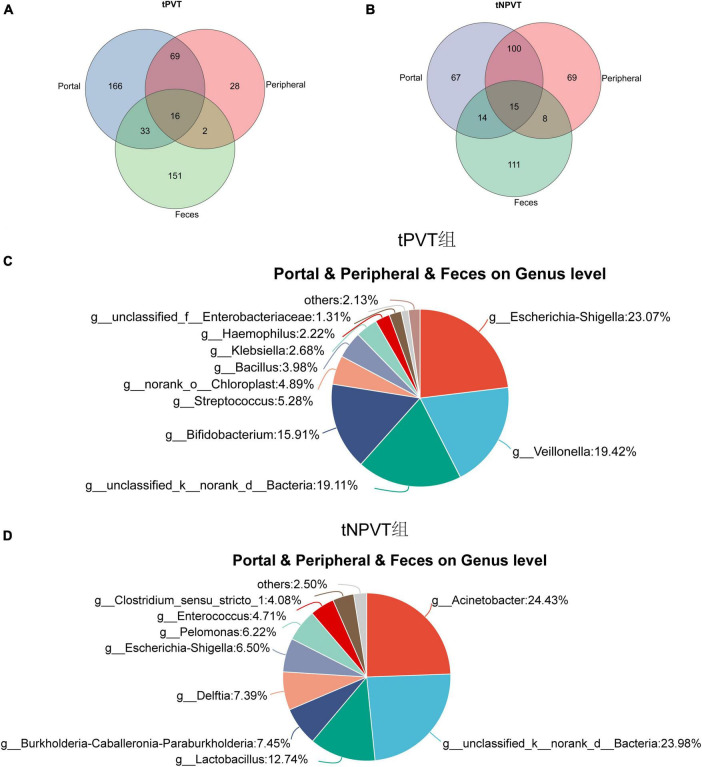
**(A)** Venn diagram of portal venous blood, peripheral venous blood, and feces at genus level bacteria in tPVT group. **(B)** Venn diagram of portal venous blood, peripheral venous blood, and feces at genus level bacteria in tNPVT group. **(C)** The distribution of bacterial genera shared among the portal vein blood, peripheral vein blood, and feces at the genus level in the tPVT group. **(D)** The distribution of bacterial genera shared among the portal vein blood, peripheral vein blood, and feces at the genus level in the tNPVT group.

The Venn diagram results show that the tPVT group shares 49 genera in the portal venous blood and feces, and 16 genera are shared among the portal venous blood, peripheral venous blood, and feces (Figure 4A). These shared genera include: *g_Escherichia-Shigella* (23.07%), *g_Veillonella* (19.42%), *g_Bifidobacterium* (15.91%), *g_Streptococcus* (5.28%), etc (Figure 4C). The tNPVT group shares 29 genera in the portal venous blood and feces, and 15 genera are shared among the portal venous blood, peripheral venous blood, and feces (Figure 4B). These shared genera include: *g_Acinetobacter* (24.43%), *g_Lactobacillus* (12.74%), *g_Delftia* (7.39%), *g_Escherichia-Shigella* (6.50%), etc (Figure 4D).

### 3.7 Heatmap analysis of the correlation between microbial communities and clinical variables

**FIGURE 5:**
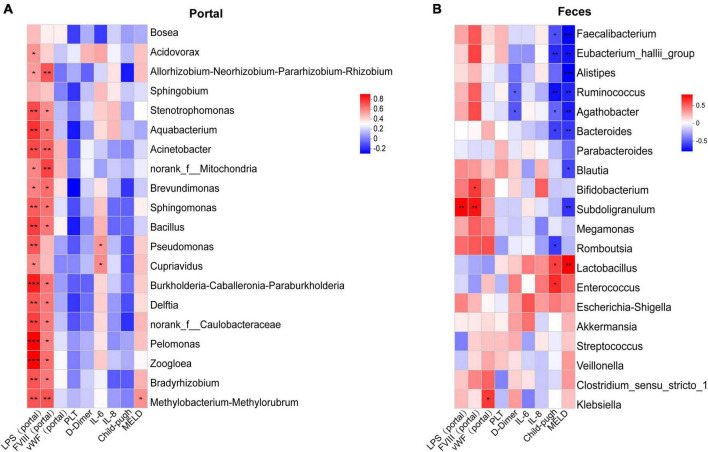
Correlation analysis of clinical indexes with different bacterial features. **(A)** Top 20 differential fecal bacterial genera most correlated with clinical indexes in tPVT groups. **(B)** 20 differential portal bacterial genera most correlated with clinical indexes in tPVT groups. The red color in the heatmap represents positive correlation and the blue color represents negative correlation. *P < 0.05; **P < 0.01; ***P < 0.001 (FDR corrected).

Using the Spearman correlation coefficient, the correlation between microbial taxa and clinical variables was evaluated. Based on the number of significant factors and the size of the *P*-values, the top twenty most highly correlated differential genera were displayed in the heatmap. Overall, several genera in the portal vein blood showed significant associations with LPS and FVIII in the portal vein (Figure 5A). Except for *Bosea* and *Sphingobium*, the remaining 18 genera were significantly positively correlated with LPS in the portal vein (*P* < 0.05). Except for *Bosea*, *Sphingobium*, *Acidovorax*, *Pseudomonas*, and *Cupriavidus*, the other 15 genera were significantly positively correlated with FVIII in the portal vein (*P* < 0.05). There were also significant associations between various genera in the intestinal microbiota and clinical parameters, as well as LPS and FVIII in the portal vein (Figure 5B). *Faecalibacterium*, *Eubacterium_hallii_group*, *Ruminococcus*, *Agathobacter*, *Bacteroides*, and *Romboutsia* were significantly negatively correlated with the Child-Pugh score (*P* < 0.05). *Faecalibacterium*, *Eubacterium_hallii_group*, *Alistipes*, *Ruminococcus*, *Agathobacter*, *Bacteroides*, *Blautia*, and *Subdoligranulum* were significantly negatively correlated with the MELD score (*P* < 0.05). *Ruminococcus* and *Agathobacter* were significantly negatively correlated with D-Dimer (*P* < 0.05). *Subdoligranulum* was significantly positively correlated with LPS and FVIII in the portal vein (*P* < 0.01).

## 4 Discussion

The gut microbiota plays a crucial role in the development and progression of PVT in cirrhosis patients (Violi et al., 2023; Pemmasani et al., 2024). However, there is limited research on the characteristics of the circulatory microbiota in cirrhotic PVT patients and its relationship with the gut microbiota and the differences between them. This study explores the changes in the microbiota in the gut, portal vein, and peripheral venous blood of cirrhotic PVT patients, as well as the interactions between these microbiota in different compartments.

This study found that the gut microbiota α-diversity in the PVT group was higher than that in the NPVT group, and there was a significant difference in β-diversity between the two groups. These findings suggest a marked dysbiosis in the gut microbiota of cirrhotic PVT patients, with significant changes in the community structure of the microbiota. Huang et al. (2023) found that in cirrhotic PVT patients, the abundance of pathogenic bacteria such as *anaerobes* and *Pseudomonas* increased, while the abundance of beneficial bacteria such as *Bacteroides* and *Weissella* significantly decreased. Another study (Georgescu et al., 2023) also found that the incidence of overall microbiota dysbiosis was increased in cirrhotic PVT patients, with a significant increase in *LPS* + *bacteria*. The results of this study found that in the gut microbiota, the abundance of Gram-negative bacilli such as *Escherichia-Shigella* and *Negativibacillus* was significantly increased in the PVT group, while beneficial bacteria such as *Bacteroides*, *Butyricicoccus*, and *Lachnospiraceae* were significantly decreased. The Shiga toxin produced by *Escherichia-Shigella* can cause endothelial cell damage, leading to endothelial dysfunction and promoting thrombus formation (Siniscalchi et al., 2024). It can also lead to excessive activation of coagulation factors, triggering a systemic coagulation response (Adams et al., 2017). Studies have found that in patients with liver cirrhosis, intestinal barrier dysfunction allows *Escherichia-Shigella* and its produced LPS to enter the portal vein through the gut-liver axis, triggering immune responses and coagulation abnormalities, thereby promoting the formation of PVT (Nightingale and Cutler, 2013). He et al. (2022) found that *Escherichia-Shigella* infection significantly reduces gut microbial diversity, increases the proportion of harmful bacteria such as *Proteus*, and decreases beneficial bacteria such as *Lactobacillus*, leading to a decrease in SCFAs levels. The reduction in SCFAs is closely associated with inflammation and coagulation abnormalities. *Negativibacillus* is a pathogenic bacterium associated with intestinal dysbiosis or pediatric Crohn’s disease (Tang et al., 2021; Wang et al., 2021). Studies have found that *Negativibacillus* is significantly negatively correlated with the length of the small intestine and colon, leading to impaired intestinal development and dysfunction of the intestinal barrier in mice (Wu et al., 2021). *Bacteroides* is an important component of the gut microbiota. It may indirectly influence thrombosis formation by regulating intestinal barrier function and immune responses (Liu et al., 2024; Khuu et al., 2025). A study by Popescu et al. (2024) found that *Bacteroides* exerts anti-inflammatory effects through the production of SCFAs, which may indirectly reduce the risk of thrombosis. Huang et al. (2023) discovered that in cirrhotic PVT patients, the abundance of *Bacteroides* significantly decreased. Their research also showed that *Bacteroides* effectively reversed the formation of PVT in a rat model of cirrhosis induced by carbon tetrachloride. In this study, *Bacteroides* was significantly reduced in the PVT group, suggesting that *Bacteroides* plays a protective role in the development of cirrhotic PVT. *Butyricicoccus* produces butyrate, which plays a crucial role in maintaining the intestinal barrier, inhibiting intestinal inflammation, promoting immune regulation in the gut, and modulating systemic immune responses. Butyrate has been shown to regulate the inflammatory response and oxidative stress in endothelial cells by inhibiting histone deacetylases (HDACs), processes that are important in thrombosis formation (Umei et al., 2021). In cirrhotic PVT patients, the gut microbiota is severely dysregulated, primarily characterized by an increase in Gram-negative pathogenic bacteria and a decrease in beneficial SCFA-producing bacteria. Modulating the gut microbiota may serve as a potential therapeutic strategy to alleviate symptoms and improve prognosis in cirrhotic PVT patients.

The dysregulated gut microbiota in cirrhosis can translocate into the bloodstream through the compromised intestinal barrier, further influencing the microbiome in the portal vein and peripheral vein. The blood microbiome is primarily dominated by Proteobacteria, with low concentrations of *Actinobacteriota*, *Bacteroidota*, and *Firmicutes*, consistent with previous studies (Iebba et al., 2018; Alvarez-Silva et al., 2019; Schierwagen et al., 2019). In this study, no significant differences were found in the α and β diversity of the peripheral venous microbiota between the PVT and NPVT groups, suggesting that the peripheral venous microbiota in cirrhotic PVT patients remains relatively stable and is not influenced by the gut microbiota. However, significant differences were observed in the α and β diversity of the portal venous microbiota. As the central pathway of the “gut-liver axis,” the microbiota of the portal vein directly reflects the effects of gut microbiota translocation, while the peripheral venous microbiota remains relatively stable due to liver filtration and systemic immune clearance. LEfSe analysis revealed that *Massilia* was significantly increased in the portal circulation of the tPVT group. *Massilia*, a Gram-negative bacterium, has been shown in a study to have a higher relative abundance in the blood microbiome of patients with cirrhosis and chronic hepatitis C, especially in those whose hepatic venous pressure gradient has not decreased, and it is significantly associated with inflammation and plasma biomarkers of metabolism (Virseda-Berdices et al., 2022). Park and Shin (2013) isolated *Massilia* from the tympanic membrane exudate of patients with otitis media. Additionally, *Massilia* has been identified in the femurs of patients with osteomyelitis, in the eyes of patients with endophthalmitis, in the cerebrospinal fluid of patients with pseudotumors, as well as in the blood of patients with cerebellar lesions, end-stage renal disease, sepsis, and common variable immunodeficiency—conditions associated with immune dysfunction (La Scola et al., 1998; Lindquist et al., 2003; Kämpfer et al., 2012). Furthermore, studies have indicated that overgrowth of *Massilia* in cases of dysbiosis may exacerbate inflammation (Aghamajidi and Maleki Vareki, 2022; Maciel-Fiuza et al., 2023). Given the commonly observed immunosuppressed state in patients with cirrhosis and PVT, we observed a significant enrichment of *Massilia* in their portal vein blood, suggesting a potential link to local inflammatory responses or the status of the coagulation system. However, there is currently no direct evidence indicating a pathogenic role of *Massilia* in the formation of PVT. Further studies incorporating functional experiments are needed to explore the potential mechanistic role and clinical significance of this genus in the pathogenesis of PVT.

The gut microbiome has been utilized as a novel molecular biomarker, with previous studies evaluating diagnostic accuracy based on liver fibrosis/cirrhosis-associated bacteria and metabolomic characteristics, yielding effective results (AUC: 0.72–0.91) (Loomba et al., 2019; Lee et al., 2020). The present study found that the AUC values of fecal microbiome and portal vein biomarkers were 0.78 and 0.95, respectively, both demonstrating good diagnostic value. In contrast, the AUC of peripheral venous biomarkers was 0.43, indicating that portal vein microbiome is the optimal biomarker for diagnosing PVT. The high AUC value highlights the crucial role of the “gut-portal vein-liver” axis in thrombosis formation. Portal vein microbiome detection shows potential clinical value in the early diagnosis of PVT. However, this analysis is exploratory in nature and the observed AUC should be interpreted with caution. The identified microbial features should be validated in larger, independent cohorts to confirm their diagnostic potential and reproducibility.

Liver diseases are closely related to gut microbiome translocation. Schierwagen et al. (2019) found strong evidence of bacterial translocation, showing that in patients with decompensated cirrhosis, the bacteria in different circulatory compartments (portal vein, hepatic vein, and central vein) are similar. Iebba et al. (2018) discovered that the microbiome composition in the portal vein blood of cirrhotic patients resembles that of the cecal mucosa, suggesting that the portal vein microbiome may originate from gut microbiome translocation. Effenberger et al. (2023) found that DNA from the fecal microbiome was significantly enriched in the blood and liver tissues of patients with liver cancer and cirrhosis, compared to patients with non-alcoholic fatty liver disease (NAFLD). The Venn diagram results of this study show that the tPVT group has more shared bacterial genera between the portal vein and feces compared to the tNPVT group, indicating a more significant abnormal translocation of gut microbiota into the portal venous system in patients with PVT. This suggests a more severe impairment of the intestinal barrier function in cirrhotic PVT patients, leading to a greater amount of gut microbiota entering the portal venous system. The shared microbiota between the portal vein, peripheral vein, and feces in the tPVT group primarily consists of pathogenic bacteria such as *Escherichia-Shigella*, *Veillonella*, *Streptococcus*, *Bacillus*, *Klebsiella*, and *Haemophilus*. *Veillonella* can work synergistically with *Streptococcus* to release endotoxins and inflammatory factors, thereby damaging the intestinal barrier function (Mashima and Nakazawa, 2014). In the gut microbiota, many facultative anaerobes (such as *Streptococcus*) can survive and proliferate in oxygen-rich living tissues. When the intestinal barrier weakens or their numbers increase, these bacteria can translocate from the intestinal contents to the intestinal wall and further spread into the bloodstream (Arab et al., 2018; Ponziani et al., 2018; Jin et al., 2022). Pathogenic bacteria and pro-inflammatory cytokines (such as TNF-α, IL-6) released from the intestinal inflammatory microenvironment can migrate to the liver through the portal venous system, exacerbating the process of liver cirrhosis and fibrosis (Arab et al., 2018; Skinner et al., 2020). The findings of this study suggest that pathogenic bacteria from the inflamed gut microenvironment may translocate to the liver via the portal venous system, potentially playing a critical role in the development and progression of PVT. Our research team plans to integrate data on gut and portal vein microbiota composition, inflammatory markers, and coagulation function to construct a personalized PVT risk assessment model, providing a basis for early clinical intervention. Strategies aimed at improving gut barrier function or modulating microbial composition—such as the use of probiotics, fecal microbiota transplantation, or targeted antibiotics—hold promise for preventing bacterial translocation and the spread of inflammatory mediators, thereby delaying or even preventing the onset of PVT. However, the feasibility and safety of these diagnostic and therapeutic approaches require further validation through large-scale clinical studies and mechanistic investigations.

Spearman rank correlation analysis was used to assess the relationship between the microbiota and clinical parameters. In the tPVT group, the majority of portal vein microbiota in portal vein blood showed significant positive correlations with LPS and FVIII. LPS can activate the NF-κB signaling pathway by binding to TLR4, prompting monocytes, endothelial cells, and other cells to release pro-inflammatory factors (such as TNF-α, IL-6, IL-1β) (Khuu et al., 2025). These inflammatory factors can activate the coagulation system, thus promoting the formation of PVT. Iebba et al. (2018) found that in cirrhotic patients, both peripheral blood and portal vein blood mainly consisted of p_Proteobacteria, and the presence of *p_Proteobacteria* members was positively correlated with the levels of IL-6, IL-1β, and TNF-α. Georgescu et al. (2023) reported a significant increase in LPS + bacteria in cirrhotic PVT patients, with a significant correlation between *LPS* + *bacteria* and biomarkers (such as D-Dimer, CRP, and TNF-α), as well as with the severity of PVT. In the study of the relationship between intestinal microbiota and blood parameters in the tPVT group, it was found that *Faecalibacterium*, *Ruminococcus*, *Bacteroides*, *Eubacterium_hallii_group*, *Agathobacter*, and *Romboutsia* were significantly negatively correlated with the Child-Pugh score, suggesting that these bacteria may have a protective role in maintaining liver metabolism and immune stability. Additionally, *Faecalibacterium*, *Ruminococcus*, *Bacteroides*, *Eubacterium_hallii_group*, *Alistipes*, *Agathobacter*, *Blautia*, and *Subdoligranulum* were negatively correlated with the MELD score, indicating that dysbiosis could be an important feature of liver function deterioration. *Subdoligranulum* was positively correlated with LPS in the portal vein, and studies have shown that intestinal bacteria *Subdoligranulum* can drive the production of systemic autoantibodies, antibody deposition at joint centers, and immune activation, thus triggering rheumatoid arthritis in at-risk populations (Chriswell et al., 2022). This genus is associated with elevated endotoxins, suggesting that it may be involved in the process of intestinal barrier dysfunction and endotoxin translocation.

This study is the first to detect gut-liver ecological dysregulation associated with cirrhosis-related PVT and elucidates the role of the intestinal and circulating microbiomes, supporting the hypothesis that microbial imbalance may promote the development of PVT. To validate these findings, future studies will expand the sample size and identify more biomarkers to assist with early diagnosis and treatment of PVT. Additionally, animal models and genomics can be utilized to investigate how microbial imbalance influences PVT formation through immune modulation, intestinal barrier dysfunction, and endotoxin translocation, further exploring the role of gut-liver axis ecological dysregulation in PVT.

## Data Availability

The original contributions presented in this study are publicly available. This data can be found here: https://www.ncbi.nlm.nih.gov/, accession number: PRJNA1269780.
