# Supplementary material for: Analysis of gut and circulating microbiota characteristics in patients with liver cirrhosis and portal vein thrombosis
Source: Front Microbiol. 2025 Jun 19;16:1597145. doi: 10.3389/fmicb.2025.1597145 (PMC12222121; doi:10.3389/fmicb.2025.1597145)
Supplement: Supplementary file 1 [file Supplementary_file_1.DOCX]

Supplementary Figures


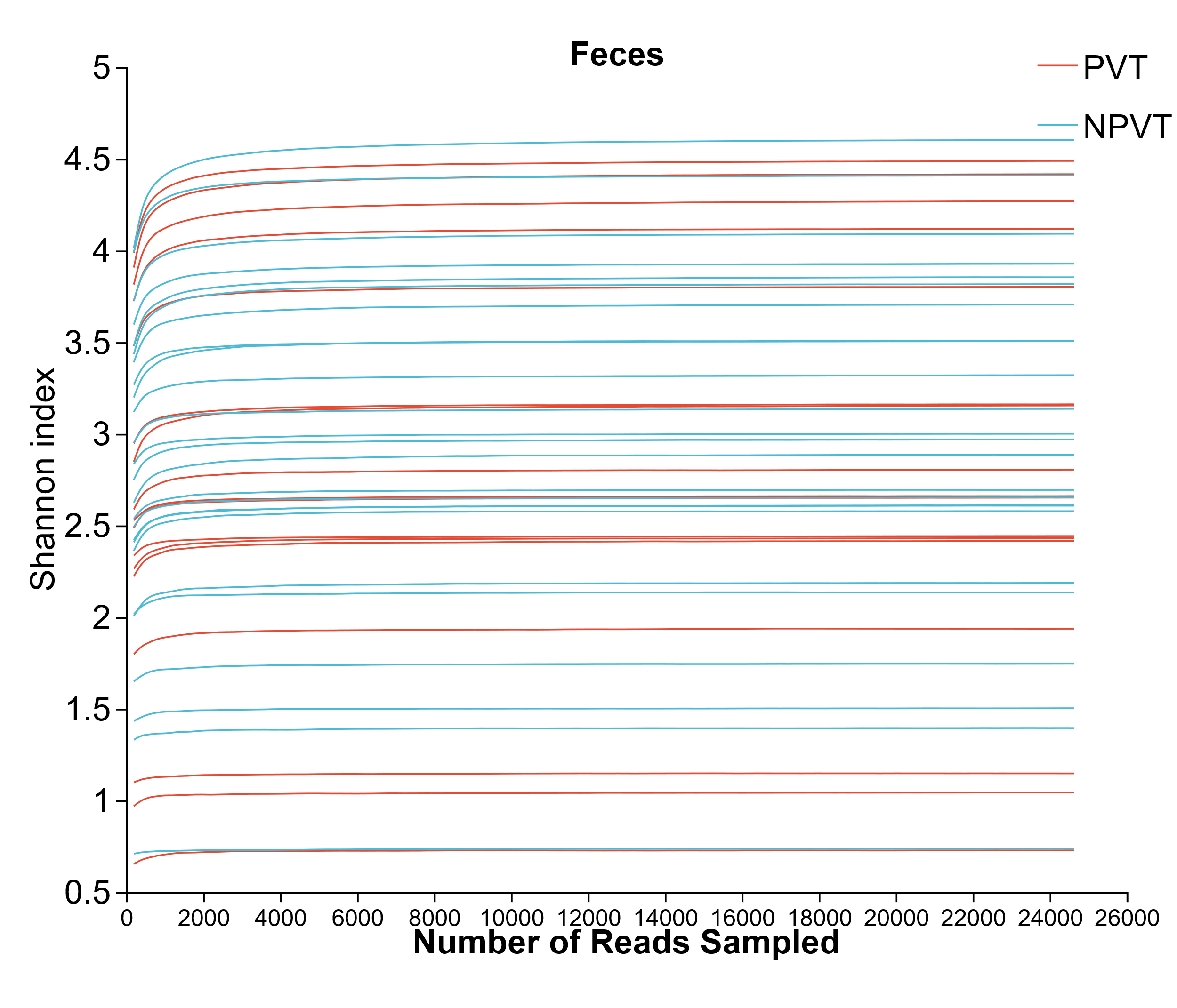


Figure 1A: Average rarefaction curves (with 95% confidence interval) based on Shannon index of feces in the PVT and NPVT groups.

Figure 1B


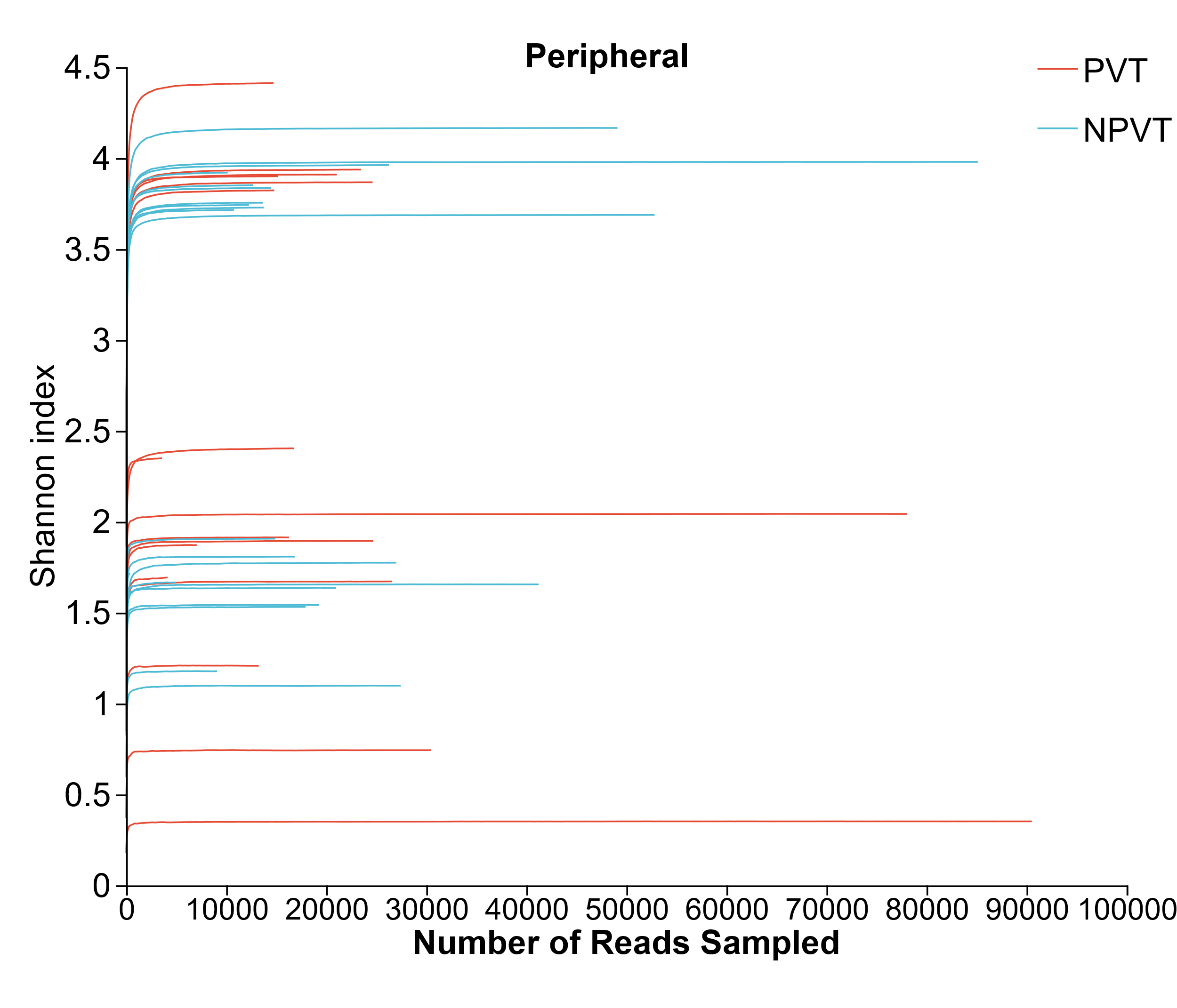


Figure 1B: Average rarefaction curves (with 95% confidence interval) based on Shannon index of peripheral venous blood in the PVT and NPVT groups.


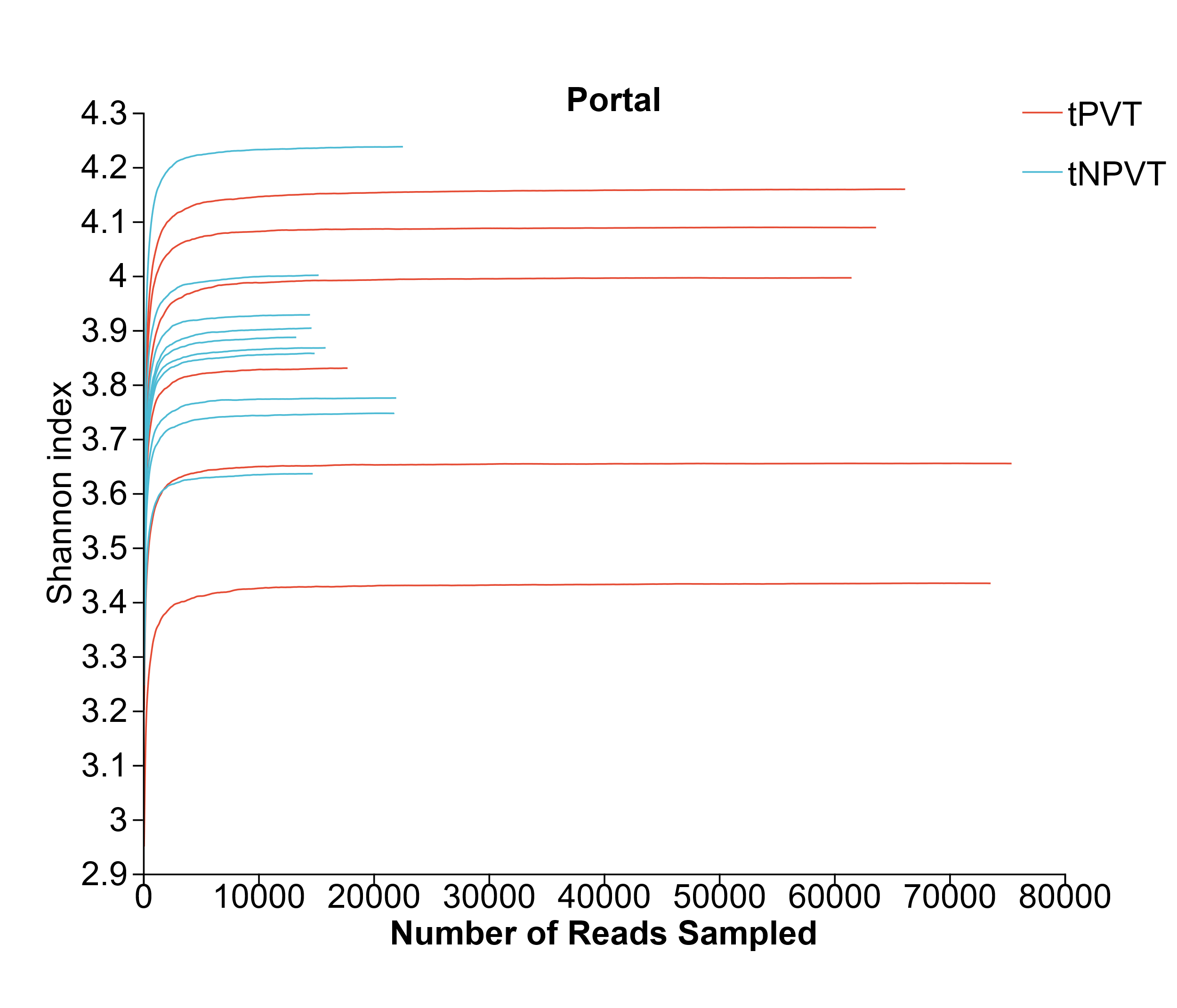


Figure 1C: Average rarefaction curves (with 95% confidence interval) based on Shannon index of portal venous blood in the tPVT and tNPVT groups.
